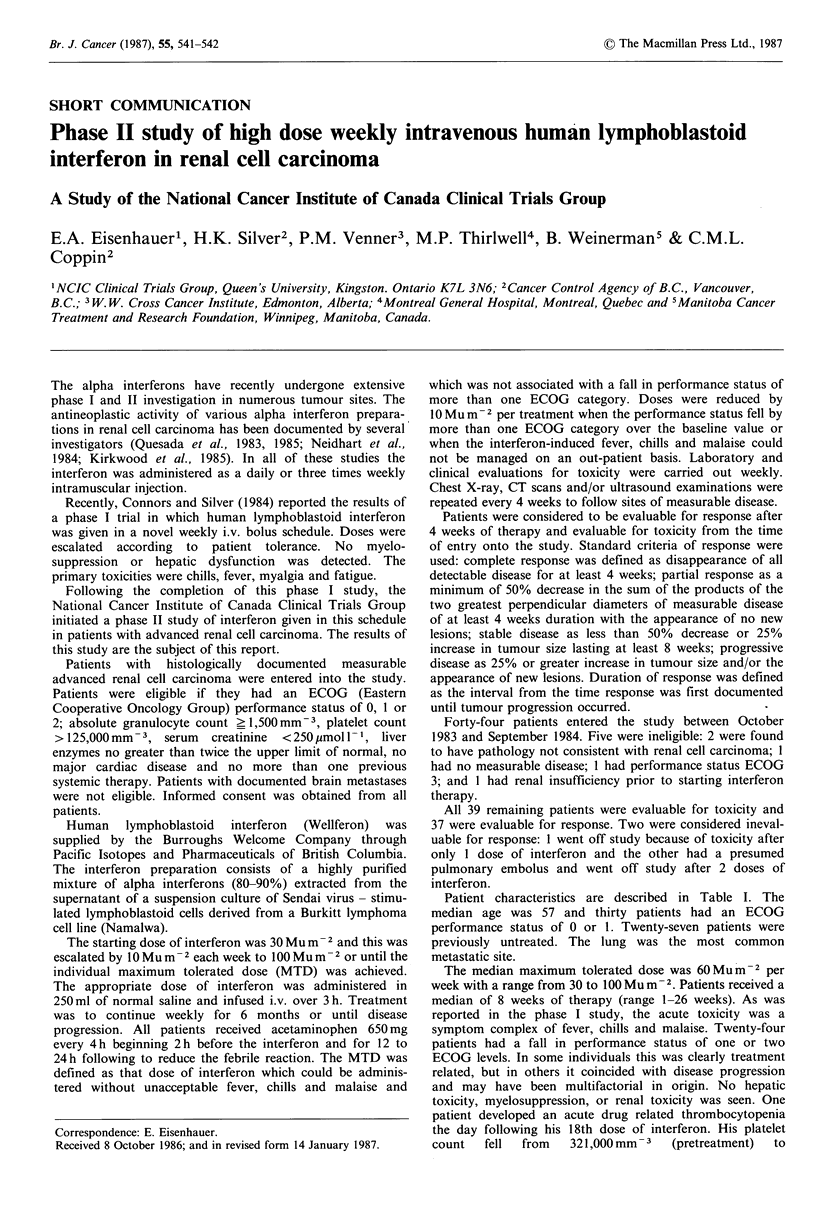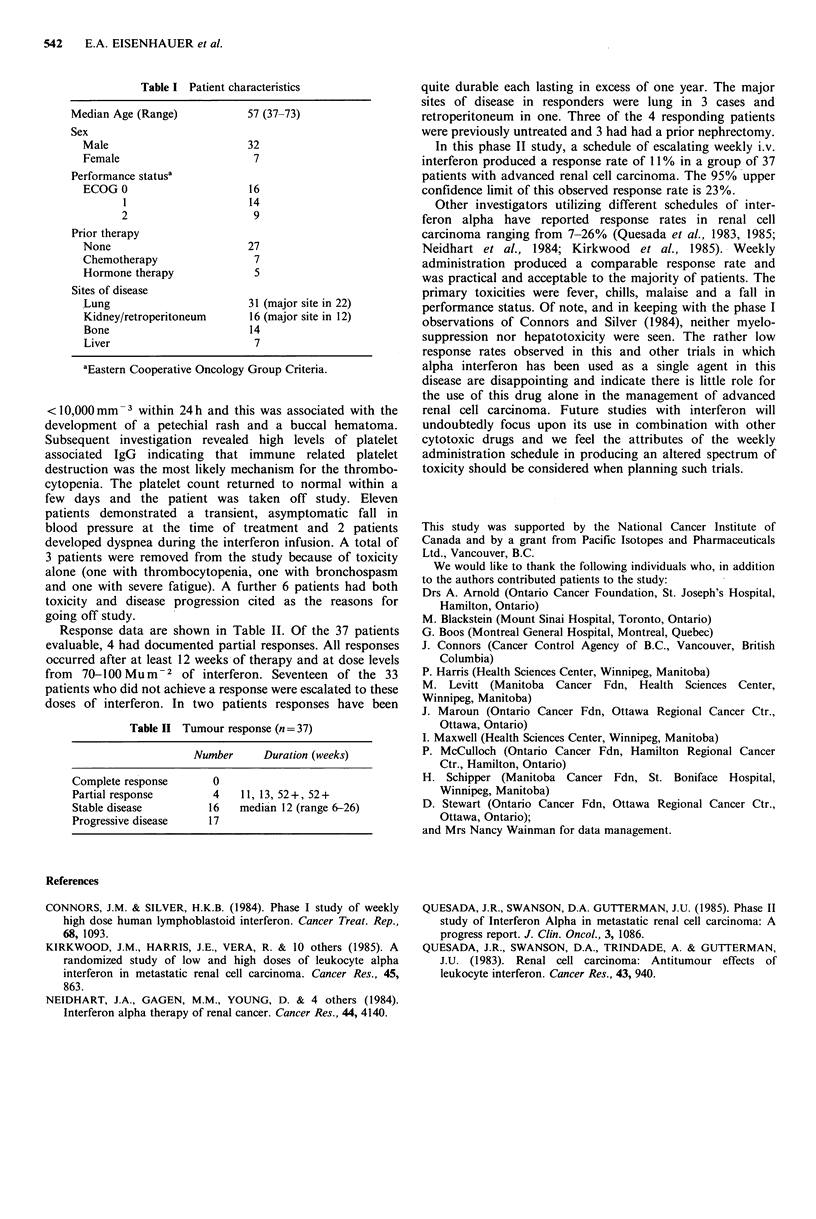# Phase II study of high dose weekly intravenous human lymphoblastoid interferon in renal cell carcinoma. A study of the National Cancer Institute of Canada Clinical Trials Group.

**DOI:** 10.1038/bjc.1987.110

**Published:** 1987-05

**Authors:** E. A. Eisenhauer, H. K. Silver, P. M. Venner, M. P. Thirlwell, B. Weinerman, C. M. Coppin


*Br. J. Cancer* (1987), **55**, 541–542 © The Macmillan Press Ltd., 1987

SHORT COMMUNICATION

### Phase II study of high dose weekly intravenous human lymphoblastoid interferon in renal cell carcinoma

### A Study of the National Cancer Institute of Canada Clinical Trials Group

E.A. Eisenhauer[1], H.K. Silver[2], P.M. Venner[3], M.P. Thirlwell[4], B. Weinerman[5] & C.M.L. Coppin[2]

[1]*NCIC Clinical Trials Group, Queen's University, Kingston. Ontario K7L 3N6;* [2]*Cancer Control Agency of B.C., Vancouver, B.C.;* [3]*W.W. Cross Cancer Institute, Edmonton, Alberta;* [4]*Montreal General Hospital, Montreal, Quebec and* [5]*Manitoba Cancer Treatment and Research Foundation, Winnipeg, Manitoba, Canada.*

The alpha interferons have recently undergone extensive phase I and II investigation in numerous tumour sites. The antineoplastic activity of various alpha interferon preparations in renal cell carcinoma has been documented by several investigators (Quesada *et al.*, 1983, 1985; Neidhart *et al.*, 1984; Kirkwood *et al.*, 1985). In all of these studies the interferon was administered as a daily or three times weekly intramuscular injection.

Recently, Connors and Silver (1984) reported the results of a phase I trial in which human lymphoblastoid interferon was given in a novel weekly i.v. bolus schedule. Doses were escalated according to patient tolerance. No myelosuppression or hepatic dysfunction was detected. The primary toxicities were chills, fever, myalgia and fatigue.

Following the completion of this phase I study, the National Cancer Institute of Canada Clinical Trials Group initiated a phase II study of interferon given in this schedule in patients with advanced renal cell carcinoma. The results of this study are the subject of this report.

Patients with histologically documented measurable advanced renal cell carcinoma were entered into the study. Patients were eligible if they had an ECOG (Eastern Cooperative Oncology Group) performance status of 0, 1 or 2; absolute granulocyte count $\geqq 1{,}500\,\mathrm{mm}^{-3}$, platelet count $>125{,}000\,\mathrm{mm}^{-3}$, serum creatinine $<250\,\mu\mathrm{mol\,l}^{-1}$, liver enzymes no greater than twice the upper limit of normal, no major cardiac disease and no more than one previous systemic therapy. Patients with documented brain metastases were not eligible. Informed consent was obtained from all patients.

Human lymphoblastoid interferon (Wellferon) was supplied by the Burroughs Welcome Company through Pacific Isotopes and Pharmaceuticals of British Columbia. The interferon preparation consists of a highly purified mixture of alpha interferons (80–90%) extracted from the supernatant of a suspension culture of Sendai virus – stimulated lymphoblastoid cells derived from a Burkitt lymphoma cell line (Namalwa).

The starting dose of interferon was $30\,\mathrm{Mu\,m}^{-2}$ and this was escalated by $10\,\mathrm{Mu\,m}^{-2}$ each week to $100\,\mathrm{Mu\,m}^{-2}$ or until the individual maximum tolerated dose (MTD) was achieved. The appropriate dose of interferon was administered in 250 ml of normal saline and infused i.v. over 3 h. Treatment was to continue weekly for 6 months or until disease progression. All patients received acetaminophen 650 mg every 4 h beginning 2 h before the interferon and for 12 to 24 h following to reduce the febrile reaction. The MTD was defined as that dose of interferon which could be administered without unacceptable fever, chills and malaise and which was not associated with a fall in performance status of more than one ECOG category. Doses were reduced by $10\,\mathrm{Mu\,m}^{-2}$ per treatment when the performance status fell by more than one ECOG category over the baseline value or when the interferon-induced fever, chills and malaise could not be managed on an out-patient basis. Laboratory and clinical evaluations for toxicity were carried out weekly. Chest X-ray, CT scans and/or ultrasound examinations were repeated every 4 weeks to follow sites of measurable disease.

Patients were considered to be evaluable for response after 4 weeks of therapy and evaluable for toxicity from the time of entry onto the study. Standard criteria of response were used: complete response was defined as disappearance of all detectable disease for at least 4 weeks; partial response as a minimum of 50% decrease in the sum of the products of the two greatest perpendicular diameters of measurable disease of at least 4 weeks duration with the appearance of no new lesions; stable disease as less than 50% decrease or 25% increase in tumour size lasting at least 8 weeks; progressive disease as 25% or greater increase in tumour size and/or the appearance of new lesions. Duration of response was defined as the interval from the time response was first documented until tumour progression occurred.

Forty-four patients entered the study between October 1983 and September 1984. Five were ineligible: 2 were found to have pathology not consistent with renal cell carcinoma; 1 had no measurable disease; 1 had performance status ECOG 3; and 1 had renal insufficiency prior to starting interferon therapy.

All 39 remaining patients were evaluable for toxicity and 37 were evaluable for response. Two were considered inevaluable for response: 1 went off study because of toxicity after only 1 dose of interferon and the other had a presumed pulmonary embolus and went off study after 2 doses of interferon.

Patient characteristics are described in Table I. The median age was 57 and thirty patients had an ECOG performance status of 0 or 1. Twenty-seven patients were previously untreated. The lung was the most common metastatic site.

The median maximum tolerated dose was $60\,\mathrm{Mu\,m}^{-2}$ per week with a range from 30 to $100\,\mathrm{Mu\,m}^{-2}$. Patients received a median of 8 weeks of therapy (range 1–26 weeks). As was reported in the phase I study, the acute toxicity was a symptom complex of fever, chills and malaise. Twenty-four patients had a fall in performance status of one or two ECOG levels. In some individuals this was clearly treatment related, but in others it coincided with disease progression and may have been multifactorial in origin. No hepatic toxicity, myelosuppression, or renal toxicity was seen. One patient developed an acute drug related thrombocytopenia the day following his 18th dose of interferon. His platelet count fell from $321{,}000\,\mathrm{mm}^{-3}$ (pretreatment) to

Correspondence: E. Eisenhauer.
Received 8 October 1986; and in revised form 14 January 1987.

**Table I** Patient characteristics

| | |
|---|---|
| Median Age (Range) | 57 (37–73) |
| Sex | |
| Male | 32 |
| Female | 7 |
| Performance status[a] | |
| ECOG 0 | 16 |
| 1 | 14 |
| 2 | 9 |
| Prior therapy | |
| None | 27 |
| Chemotherapy | 7 |
| Hormone therapy | 5 |
| Sites of disease | |
| Lung | 31 (major site in 22) |
| Kidney/retroperitoneum | 16 (major site in 12) |
| Bone | 14 |
| Liver | 7 |

[a]Eastern Cooperative Oncology Group Criteria.

$<10{,}000\,mm^{-3}$ within 24 h and this was associated with the development of a petechial rash and a buccal hematoma. Subsequent investigation revealed high levels of platelet associated IgG indicating that immune related platelet destruction was the most likely mechanism for the thrombocytopenia. The platelet count returned to normal within a few days and the patient was taken off study. Eleven patients demonstrated a transient, asymptomatic fall in blood pressure at the time of treatment and 2 patients developed dyspnea during the interferon infusion. A total of 3 patients were removed from the study because of toxicity alone (one with thrombocytopenia, one with bronchospasm and one with severe fatigue). A further 6 patients had both toxicity and disease progression cited as the reasons for going off study.

Response data are shown in Table II. Of the 37 patients evaluable, 4 had documented partial responses. All responses occurred after at least 12 weeks of therapy and at dose levels from 70–100 $Mu\,m^{-2}$ of interferon. Seventeen of the 33 patients who did not achieve a response were escalated to these doses of interferon. In two patients responses have been quite durable each lasting in excess of one year. The major sites of disease in responders were lung in 3 cases and retroperitoneum in one. Three of the 4 responding patients were previously untreated and 3 had had a prior nephrectomy.

**Table II** Tumour response ($n=37$)

| | *Number* | *Duration (weeks)* |
|---|---|---|
| Complete response | 0 | |
| Partial response | 4 | 11, 13, 52+, 52+ |
| Stable disease | 16 | median 12 (range 6–26) |
| Progressive disease | 17 | |

In this phase II study, a schedule of escalating weekly i.v. interferon produced a response rate of 11% in a group of 37 patients with advanced renal cell carcinoma. The 95% upper confidence limit of this observed response rate is 23%.

Other investigators utilizing different schedules of interferon alpha have reported response rates in renal cell carcinoma ranging from 7–26% (Quesada *et al.*, 1983, 1985; Neidhart *et al.*, 1984; Kirkwood *et al.*, 1985). Weekly administration produced a comparable response rate and was practical and acceptable to the majority of patients. The primary toxicities were fever, chills, malaise and a fall in performance status. Of note, and in keeping with the phase I observations of Connors and Silver (1984), neither myelosuppression nor hepatotoxicity were seen. The rather low response rates observed in this and other trials in which alpha interferon has been used as a single agent in this disease are disappointing and indicate there is little role for the use of this drug alone in the management of advanced renal cell carcinoma. Future studies with interferon will undoubtedly focus upon its use in combination with other cytotoxic drugs and we feel the attributes of the weekly administration schedule in producing an altered spectrum of toxicity should be considered when planning such trials.

This study was supported by the National Cancer Institute of Canada and by a grant from Pacific Isotopes and Pharmaceuticals Ltd., Vancouver, B.C.

We would like to thank the following individuals who, in addition to the authors contributed patients to the study:

Drs A. Arnold (Ontario Cancer Foundation, St. Joseph's Hospital, Hamilton, Ontario)

M. Blackstein (Mount Sinai Hospital, Toronto, Ontario)

G. Boos (Montreal General Hospital, Montreal, Quebec)

J. Connors (Cancer Control Agency of B.C., Vancouver, British Columbia)

P. Harris (Health Sciences Center, Winnipeg, Manitoba)

M. Levitt (Manitoba Cancer Fdn, Health Sciences Center, Winnipeg, Manitoba)

J. Maroun (Ontario Cancer Fdn, Ottawa Regional Cancer Ctr., Ottawa, Ontario)

I. Maxwell (Health Sciences Center, Winnipeg, Manitoba)

P. McCulloch (Ontario Cancer Fdn, Hamilton Regional Cancer Ctr., Hamilton, Ontario)

H. Schipper (Manitoba Cancer Fdn, St. Boniface Hospital, Winnipeg, Manitoba)

D. Stewart (Ontario Cancer Fdn, Ottawa Regional Cancer Ctr., Ottawa, Ontario); and Mrs Nancy Wainman for data management.

### References

CONNORS, J.M. & SILVER, H.K.B. (1984). Phase I study of weekly high dose human lymphoblastoid interferon. *Cancer Treat. Rep.*, **68**, 1093.

KIRKWOOD, J.M., HARRIS, J.E., VERA, R. & 10 others (1985). A randomized study of low and high doses of leukocyte alpha interferon in metastatic renal cell carcinoma. *Cancer Res.*, **45**, 863.

NEIDHART, J.A., GAGEN, M.M., YOUNG, D. & 4 others (1984). Interferon alpha therapy of renal cancer. *Cancer Res.*, **44**, 4140.

QUESADA, J.R., SWANSON, D.A. GUTTERMAN, J.U. (1985). Phase II study of Interferon Alpha in metastatic renal cell carcinoma: A progress report. *J. Clin. Oncol.*, **3**, 1086.

QUESADA, J.R., SWANSON, D.A., TRINDADE, A. & GUTTERMAN, J.U. (1983). Renal cell carcinoma: Antitumour effects of leukocyte interferon. *Cancer Res.*, **43**, 940.